# HARNESSING ADVANCES IN ARTIFICIAL INTELLIGENCE TO SUPPORT AN AGING POPULATION

**DOI:** 10.1093/geroni/igae098.0910

**Published:** 2024-12-31

**Authors:** Walter Boot, Xin Yao Lin

**Affiliations:** Chair; Co-Chair

## Abstract

We are witnessing unprecedented growth in the sophistication of artificial intelligence (AI) and its rapid diffusion into all aspects of our lives. This session will focus on the potential and pitfalls of using AI to support the health, wellbeing, and quality of life of older people. Dr. Clara Berridge will contribute a big-picture discussion of outstanding ethical issues in AI, including large language model (LLM) tools, and provide an organized framework to promote the benefits of AI while minimizing ethical concerns. Dr. Shelia Cotten will discuss the integration of AI in healthcare, focusing on stakeholder acceptance and ethical concerns, and present suggestions to advance AI acceptance based on a scoping review of AI-enabled devices in diagnosis, treatment, and rehabilitation settings. Dr. Catherine Diaz-Asper will address the ethical challenges of language technologies in AI, highlighting issues of transparency, equity, and bias, and propose methods to boost user trust, particularly among older adults, through stakeholder co-design and user-friendly interfaces. Zhimeng Luo, MS, will explore the capabilities of large multimodal models (LMMs) to interpret healthcare infographics for dementia caregivers, demonstrating their accuracy and completeness and suggesting implications for content generation and health education material design. Dr. Lin Jiang will examine the use of artificial intelligence in supporting Alzheimer’s and dementia family caregivers, presenting findings from mixed-methods research that evaluates AI’s role in providing emotional and social support, and discussing the importance of interdisciplinary collaboration and caregiver involvement in AI development.

